# YOLO-HVS: Infrared Small Target Detection Inspired by the Human Visual System

**DOI:** 10.3390/biomimetics10070451

**Published:** 2025-07-08

**Authors:** Xiaoge Wang, Yunlong Sheng, Qun Hao, Haiyuan Hou, Suzhen Nie

**Affiliations:** 1 School of Mechanical Engineering, Shandong University of Technology, Zibo 255000, China; wangxg124@163.com (X.W.); houhaiyuan11@163.com (H.H.); szhen0203@163.com (S.N.); 2 Changchun University of Science and Technology, Changchun 130022, China; qhao@bit.edu.cn

**Keywords:** infrared small target detection, human visual system, YOLOv8, attention mechanism

## Abstract

To address challenges of background interference and limited multi-scale feature extraction in infrared small target detection, this paper proposes a YOLO-HVS detection algorithm inspired by the human visual system. Based on YOLOv8, we design a multi-scale spatially enhanced attention module (MultiSEAM) using multi-branch depth-separable convolution to suppress background noise and enhance occluded targets, integrating local details and global context. Meanwhile, the C2f_DWR (dilation-wise residual) module with regional-semantic dual residual structure is designed to significantly improve the efficiency of capturing multi-scale contextual information by expanding convolution and two-step feature extraction mechanism. We construct the DroneRoadVehicles dataset containing 1028 infrared images captured at 70–300 m, covering complex occlusion and multi-scale targets. Experiments show that YOLO-HVS achieves mAP50 of 83.4% and 97.8% on the public dataset DroneVehicle and the self-built dataset, respectively, which is an improvement of 1.1% and 0.7% over the baseline YOLOv8, and the number of model parameters only increases by 2.3 M, and the increase of GFLOPs is controlled at 0.1 G. The experimental results demonstrate that the proposed approach exhibits enhanced robustness in detecting targets under severe occlusion and low SNR conditions, while enabling efficient real-time infrared small target detection.

## 1. Introduction

Infrared small target detection (IRSTD) utilizes thermal radiation discrepancies between targets and their backgrounds in infrared imaging to identify and track objects of interest. Due to its strong penetration, good concealment, strong anti-jamming ability, and independence of light conditions, it has found extensive applications across multiple domains, including military operations, surveillance systems, early warning mechanisms, precision targeting, and maritime reconnaissance [1,2]. Compared with ordinary visible targets, infrared weak target imaging distance is long, resulting in the target pixels accounting for a small proportion of the pixels of the entire image and the lack of obvious texture, shape, and structure features [3,4]. Secondly, the energy of infrared radiation decays significantly with the increase of distance, which makes the infrared weak targets easily submerged in the background clutter and noise. The detection of infrared small targets remains technically challenging in contemporary research due to the extremely low pixel values of the small targets, their extremely low contrast, and the extremely close proximity of the small targets. In addition, real-time target output is required in many application scenarios, so detection algorithms with fast detection speed and a low number of model parameters are more popular [5].

With the development of deep convolutional neural networks, the performance of infrared small target detectors has been significantly enhanced. Many high-performance infrared small target detection algorithms based on deep learning have been put forward [6,7]. In general, these algorithms can be categorized into two branches. One branch of deep learning-based infrared small target detection algorithms employs a two-stage neural network as both a feature extractor and classifier, enabling the detection of infrared small targets from coarse to fine scales. Although the two-stage detector has achieved great success, it also suffers from disadvantages such as difficulty in training and slow detection speed [8]. Another branch is single-stage detection algorithms, which improve the detection speed by performing classification and regression directly on the feature map. Representatives of single-stage detection algorithms, such as YOLO [9,10], reduce the computational complexity by simplifying the detection process, thus significantly increasing the detection speed while maintaining higher accuracy [11,12].

Although deep convolutional networks significantly improve IR small target detection, detecting IR small targets with large variations in scale, occlusion, appearance, and illumination in real-world scenes is still a great challenge. Recent studies in infrared small target detection have incorporated functional characteristics inspired by the human visual system (HVS) to improve detection performance, encompassing size adaptation dynamics, contrast perception mechanisms, and attentional modulation processes. These introductions notably improve key detection capabilities, ranging from identification accuracy to computational efficiency [13,14,15]. As exemplified by the attention mechanism, its design originates from the human visual system’s selective attention in information processing: humans tend to prioritize task-relevant information when dealing with abundant data [16]. In deep learning, the attention mechanism enhances model performance by dynamically allocating higher weights to salient input features while suppressing less relevant ones, moving beyond uniform treatment of all inputs. Ref. [17] repaired the Null Convolutional Block Attention Module (DILATED CBAM) to YOLOv4’s backbone network, CSPDarknet53, to augment the small targets and make key features distinguishable. Ref. [18] proposed a dilated convolution-based weighted feature pyramid network (DWFPN). The DWFPN assigns fusion weights to multi-level features via an attention mechanism, facilitating high-quality information interaction while preventing the loss of small target features and critical information during network deepening.

Although a range of techniques have been utilized for studying infrared small target detection from different angles, the existing research still faces inherent constraints that need to be addressed [19]. On the one hand, the interference of background information is an important issue during IR small target detection. In operational scenarios, targets are commonly overshadowed by environmental noise—including vegetation, shadows, and other surface features [20]. Background interference significantly degrades detection performance by introducing false positives (misclassifying negative samples as targets), thereby compromising model accuracy. Furthermore, existing advanced methods struggle with multi-scale target extraction due to the substantial size variations typical of infrared small targets, leading to unreliable detection across different scales [21,22].

Aiming to resolve the identified limitations, this research proposes a target detection algorithm termed YOLO-HVS based on YOLOv8 and HVS. Firstly, for the purpose of reducing the impact of non-relevant information obscuring small IR targets in the detection process, the MultiSEAM (Multi-scaled Spatially Enhanced Attention Module) attention mechanism is introduced. Secondly, in order to expand the sensing field to extract rich multi-scale contextual information, C2f_DWR is designed to replace the C2f module in YOLOv8. This paper makes the following main contributions:

(1) This study constructs a multi-scenario infrared vehicle dataset containing occluded and multi-scale targets. We propose YOLO-HVS to enhance small vehicle detection, validated on both DroneVehicle and our DroneRoadVehicles datasets.

(2) To address IR small target occlusion, we integrate MultiSEAM into YOLOv8. This attention mechanism enhances unoccluded features through multi-branch depth-wise convolutions, capturing both local details and global context for robust detection.

(3) To enhance multi-scale context extraction, we propose the C2f_DWR module (C2f with dilation-wise residual) as a replacement for YOLOv8’s original C2f. This dual-residual structure splits feature extraction into regional and semantic pathways, simplifying multi-scale mapping while improving detection efficiency across varying target scales.

## 2. Datasets

The two datasets used for the experiments in this paper are now described.

### 2.1. Public Dataset-DroneVehicle

The DroneVehicle dataset [23], a comprehensive collection of aerial vehicle images captured by UAVs and publicly released by Tianjin University, serves as a critical benchmark for advancing computer vision research in unmanned aerial systems. The dataset covers rich RGB and infrared image resources for vehicle detection and counting tasks and covers diverse scenes from day to night. Given that the research focus of this paper lies in the domain of infrared small targets, only the infrared images within the dataset are employed as research objects.

In the experimental design, we strictly adhere to the public dataset partitioning strategy and partition the dataset into a training set and a validation set. Specifically, the training set is composed of 17,990 images, while the validation set includes 1469 images. The dataset encompasses five primary classes: car, truck, bus, van, and freight car. These classes cover typical vehicle types in UAV aerial photography scenarios, offering abundant sample support for infrared small target detection tasks.

### 2.2. Our Dataset-DroneRoadVehicles

It is observed that the vehicle targets within the DroneVehicle dataset exhibit relatively uniform sizes, which may be due to the relatively fixed flight altitude of the UAVs during the filming process. However, in practical applications, the flight altitude of the UAV often varies depending on the mission requirements, and this single-view shooting condition may be difficult to generalize in real-world scenarios [24]. To validate the versatility and stability of the proposed approach across varied environmental conditions, a novel dataset named DroneRoadVehicles was developed. This dataset spans an altitude range of 70 m to 300 m by capturing images at distinct flight heights, thereby encompassing large, medium, and small vehicle targets with diverse size distributions. Such a design not only augments dataset diversity but also furnishes more challenging samples for infrared small target detection tasks, enabling the validation of the method’s effectiveness and generalizability in complex scenarios.

The data collection device employed is the DJI MAVIC2 Enterprise Advanced, with its thermal and visible camera specifications fundamentally outlined in Table 1.

During infrared vehicle data acquisition, significant efforts were made to account for real-world environmental interference. The collected data span multiple diurnal cycles (daytime to nighttime) and diverse meteorological conditions (e.g., clear skies and fog), ensuring comprehensive environmental coverage. Furthermore, the dataset incorporates a wide range of practical scenarios, including but not limited to expressways, parking facilities, commercial centers, and urban crossroads. This design ensures that the dataset is diverse in terms of scenarios and environments, thus providing strong support for validating the adaptability and robustness of various types of network models under complex conditions.

**Figure 1:**
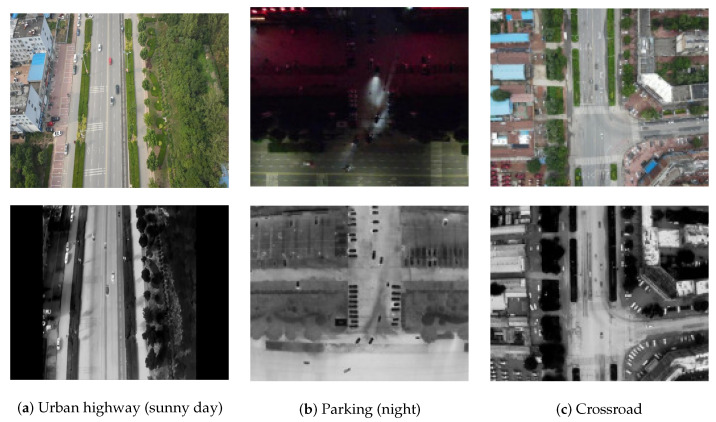
Multi-scene examples of our DroneRoadVehicles dataset.

**Figure 2:**
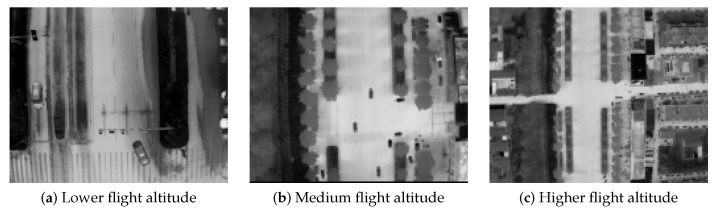
Comparison of drones at different flight altitudes.

**Figure 3:**
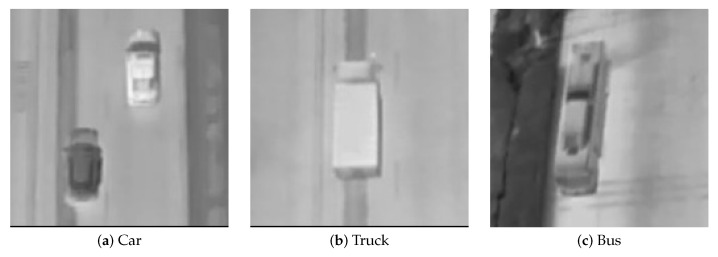
Target vehicle categories in the DroneRoadVehicles dataset.

The DroneRoadVehicles dataset contains 1028 aligned infrared-visible image pairs, divided into training (80%), validation (10%), and test sets (10%) following a standardized data split protocol. As illustrated in Figure 1, representative samples demonstrate multimodal scene coverage, where visible-spectrum images (top row) are precisely registered with their thermal infrared equivalents (bottom row) across varied environments. Figure 2 presents UAV-captured images at varying flight altitudes, further highlighting the dataset’s target size diversity. As illustrated in Figure 3, the dataset’s primary categories encompass cars, trucks, and buses. The diversity of scenes and environments within this dataset facilitates the testing of infrared vehicle target detection and tracking tasks.

## 3. Methods

### 3.1. YOLO-HVS

**Figure 4:**
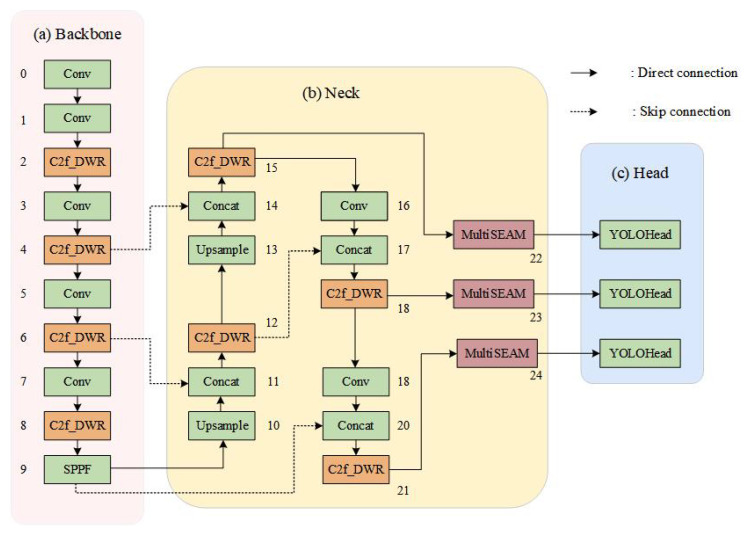
Overall network architecture of YOLO-HVS.

YOLO-HVS represents an enhanced detection architecture built upon YOLOv8, specifically tailored for infrared small object detection tasks [25]. The overall framework is visualized in Figure 4. During the optimization of the baseline YOLOv8, the following critical strategies were implemented:

First, three MultiSEAM attention mechanisms are introduced at the end of Neck. This mechanism effectively addresses the issue of inaccurate localization arising from target feature loss due to obstruction by surrounding objects. The attention module is designed to enhance target feature learning; meanwhile, it improves feature map resolution, thereby boosting detection precision for small infrared targets.

Second, we innovatively adopt C2f_DWR to replace the original C2f in YOLOv8. This improvement effectively expands the receptive field and significantly reduces the difficulty of mapping multi-scale contextual information. The expanded receptive field enables the model to integrate contextual information across broader spatial regions, thereby improving its ability to analyze target-environment interactions. Simultaneously, easing the challenge of extracting multi-scale contextual information enables the model to handle targets across diverse scales more adeptly. This optimization significantly improves the model’s capacity to detect targets across varying scales, allowing for more precise localization and identification of small infrared targets across various scales.

### 3.2. MultiSEAM Attention Mechanism

**Figure 5:**
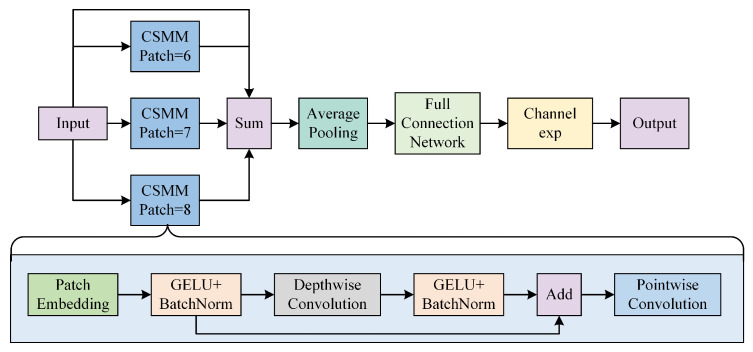
The MultiSEAM architecture (top) and its Channel-Spatial Mixing Module (CSMM, down).

In target detection, it often occurs that the target is occluded by other objects, which can lead to the disappearance of features, thus leading to inaccurate localization, and at the same time, taking into account the characteristics of infrared small target detection, we innovatively introduced a multi-branching SEAM (Self-Ensembling Attention Mechanism), known as MultiSEAM [26]. As shown in Figure 5 for the schematic diagram of the MultiSEAM Attention Mechanism.

The core design of the MultiSEAM module is shown in Figure 5, which demonstrates a multi-branch parallel architecture consisting of three CSMMs (Contextual Spatial-Channel Modeling Module) of different sizes (patch = 6/7/8) on the top side. In the feature processing flow, the input image is first segmented into multi-scale local regions (6 × 6, 7 × 7, and 8 × 8), which are mapped to a high-dimensional space by a Patch Embedding layer with shared weights to generate the initial feature representation. Each CSMM module models joint spatial-channel correlations through a two-stage operation. (1) Depthwise convolution is when the spatial features of each channel are extracted independently using depthwise convolution [27], which captures the local context information using a 3 × 3 convolution kernel and enhances the nonlinear representation through the GELU activation function with BatchNorm. (2) Channel interaction is when the spatial features of each channel are extracted independently using depthwise convolution; expression ability. (3) Channel interaction enhancement: To avoid the problem of weakening inter-channel relationships caused by deep convolution, pointwise convolution (1 × 1 convolution) is introduced to dynamically fuse cross-channel information and reconstruct the channel dependency. Further, the contextual correlation between occluded and visible regions is explicitly modeled by global channel feature fusion of multi-branch outputs through a two-layer fully connected network. Finally, the channel-wise exponential scaling (ES) features are multiplied element-by-element with the original input features to achieve feature compensation and semantic enhancement of occluded targets. This design significantly improves target recognizability in complex occlusion scenarios through multi-granularity local sensing and channel-wise adaptive feature selection mechanism.

Targeting the key challenges of minute target dimensions, low signal-to-noise ratio, and background clutter interference in infrared small target detection, MultiSEAM delivers performance advancements through multi-scale context fusion and channel attention co-optimization. In the spatial dimension, CSMM branches of different sizes focus on local details (e.g., patch = 6 to capture edge texture) and global context (e.g., patch = 8 to model long-range dependency) to form complementary multi-scale feature representations; in the channel dimension, the feature recalibration mechanism based on the attention weight (exponential expansion after Sigmoid activation) suppresses the background thermal noise channel and, at the same time, reinforces the key channel response related to the target. This feature enables robust detection performance in low illumination and strong occlusion infrared scenes, providing an efficient solution for real-time detection of small targets in complex environments.

### 3.3. C2f_DWR

Many recent methods have designed specialized backbones for real-time target detection tasks, of which the design of the receptive field is an important part [28,29]. Generally, these approaches seek a wide receptive field to acquire more contextual information, thereby enhancing feature representation. However, in practice, this may lead to inefficient feature extraction. Determining the appropriate size of the receptive field is crucial for improving the efficiency of feature extraction, and the requirement of the receptive field size varies at different stages of the network. With the enhancement of feature semantic representation, larger receptive fields are demanded at higher network stages, particularly for infrared small target detection. This study introduces novel improvements to the core modules of the YOLOv8 network, aiming to efficiently acquire multi-scale contextual information in infrared small target detection tasks. As the critical feature extraction component in target detection networks, the original C2f module realizes cross-layer feature fusion through a cascaded bottleneck structure, but its fixed-size 3 × 3 standard convolution has limitations in multi-scale feature extraction in complex contexts. Therefore, our method adopts DWR (dilation-wise residual) instead of bottleneck to compose C2f_DWR for IR small target detection.

**Figure 6:**
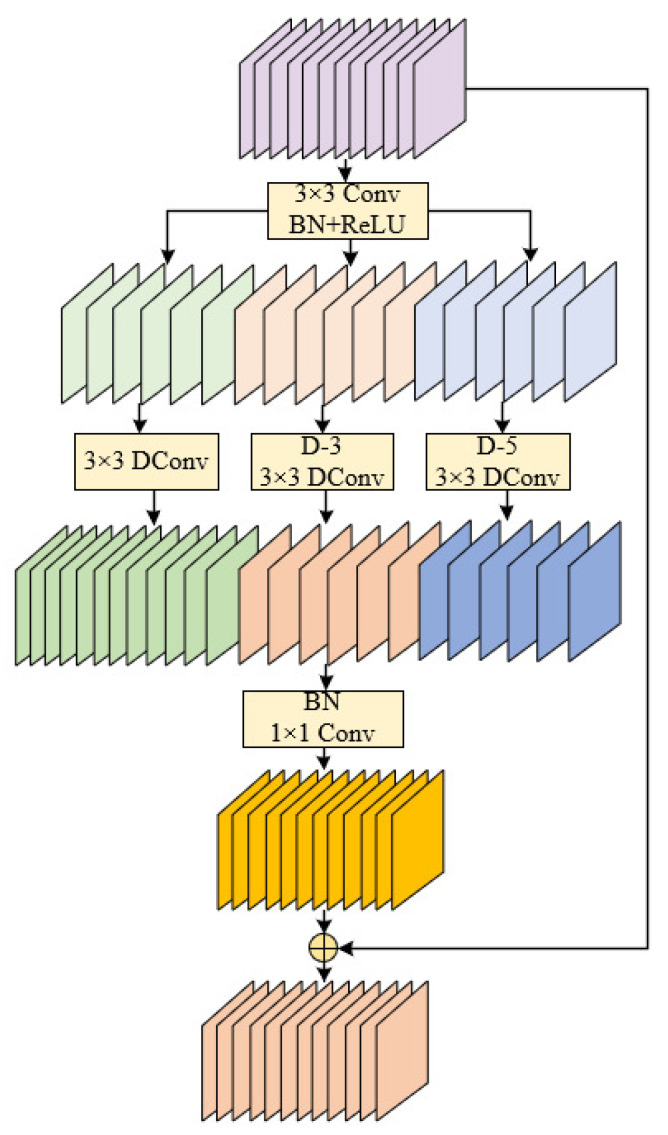
The network structure of the DWR module, where Conv represents convolution, DConv signifies depthwise convolution, D-n denotes dilated convolution with a dilation rate of n, and the circled ‘+’ indicates an addition operation.

As depicted in Figure 6, the DWR module employs an innovative dual-phase feature extraction strategy to resolve the critical challenge of receptive field expansion while preserving computational efficiency for real-time detection tasks. This design enables comprehensive contextual feature integration without compromising inference speed. This approach decomposes traditional single-step multi-scale feature extraction into two sequential processes: region residualization and semantic residualization. First, region residualization involves generating compact feature maps with distinct regional representations through 3 × 3 convolution integrated with batch normalization (BN) and ReLU activation, which establishes the foundation for morphological filtering in the subsequent step. Subsequent semantic residualization refers to semantically based morphological filtering using a depth-separable convolution of a single desired receptive field for each region feature map, avoiding unnecessary redundant receptive fields. This module employs a two-phase residual feature extraction approach (regional residualization-semantic residualization) to effectively enhance the efficiency of multi-scale information acquisition.

## 4. Experiments and Results

This section first introduces the evaluation metrics and implementation details. To demonstrate the superiority of our approach, we then conduct comparative evaluations on both the DroneVehicle and DroneRoadVehicles benchmarks. Finally, extensive ablation studies validate the individual contributions of the MultiSEAM and C2f_DWR modules.

### 4.1. Evaluation Metrics

Target detection algorithms employ multiple evaluation metrics that assess algorithm performance from diverse perspectives. The evaluation employs five key metrics: detection accuracy (precision, recall), overall performance (mean average precision), and computational efficiency (model parameters, FLOPs).

#### 4.1.1. Precision and Recall

The confusion matrix, a tabular format for evaluating classification model performance [30], compares model predictions against true labels and categorizes them into four scenarios: true positives (TP), true negatives (TN), false positives (FP), and false negatives (FN). As demonstrated in Table 2, the matrix rows denote actual categories, while columns represent predicted categories.

Precision quantifies the model’s ability to correctly identify positive instances, measured as the ratio of true positives (TP) to all predicted positives (TP + FP). In object detection, a prediction is considered valid only when the detected bounding box meets specific overlap criteria (e.g., IoU threshold) with the ground-truth annotation. The mathematical formulation is given by Equation (1).

(1) $$P=\frac{\mathrm{TP}}{\mathrm{TP}+\mathrm{FP}}$$

Recall measures the model’s coverage of positive instances, calculated as the ratio of true positives (TP) to all actual positives (TP + FN). For object detection, a ground-truth bounding box is considered successfully recalled if it matches with any predicted box meeting the predefined Intersection-over-Union (IoU) criterion. The formal definition is provided in Equation (2).

(2) $$R=\frac{\mathrm{TP}}{\mathrm{TP}+\mathrm{FN}}$$

The F1 score, defined as the harmonic mean of precision and recall, provides a balanced assessment of classification performance by equally weighting both Type I (false positives) and Type II (false negatives) errors. The calculation formula is provided in (3). Typically ranging between 0 and 1, the F1 score signifies an ideal classifier at 1 and the poorest performance at 0. This metric is especially valuable in scenarios involving imbalanced categories, as it accounts for both false positives and false negatives rather than relying solely on accuracy. In many cases, a trade-off often exists between precision and recall, and the F1 score aids in identifying an optimal balance where the classifier demonstrates robust performance across both metrics.

(3) $$F1=\frac{2\times R\times P}{R+P}$$

#### 4.1.2. mAP50 and mAP50-95

Average precision (AP) and its extension, mean average precision (MAP), constitute fundamental performance measures for object detection and semantic segmentation tasks, providing rigorous quantification of localization accuracy across all relevant object categories. Average precision (AP) serves as a fundamental performance metric in object detection, obtained by integrating precision values across all recall levels for individual object categories. This evaluation is conducted through the precision–recall (PR) curve analysis, where precision measurements are systematically recorded at incrementally varying recall thresholds. The definitive AP score is subsequently derived by computing the total area beneath this PR curve (commonly termed AUC-PR to distinguish it from ROC curve analysis), with this integration process effectively consolidating both detection accuracy and completeness into a single quantitative measure. The specific formula is

(4) $$\mathrm{AP}=\int_{0}^{1}p\left(r\right)\;dr$$

where p(r) denotes the precision at recall level *r*.

The mean average precision (mAP) serves as the primary evaluation metric by computing the arithmetic mean of category-wise AP values, thereby offering a holistic measure of model performance across all target classes. This aggregated metric is mathematically expressed in Equation (5).

(5) $$\mathrm{mAP}=\frac{1}{n}\sum_{i=1}^{n}\int_{0}^{1}p\left(r\right)\;dr$$

#### 4.1.3. Parameters and GFLOPs

Beyond task-specific performance metrics (e.g., accuracy), model efficiency metrics like parameter count and operational complexity must also be considered. The parameter count, denoting the number of model parameters, reflects the memory required for storage; operational complexity is typically measured in FLOPs (floating point operations), characterizing the computational resources needed for model inference. It is noted that FLOPs refer to the count of floating-point operations, commonly quantified in GFLOPs ($10^{9}$).

### 4.2. Experimental Setup

We use YOLOv8 as our baseline, and the method is implemented in PyTorch 1.12 using a GPU that is an NVIDIA GeForce RTX 3080Ti. In the experiments, the batch size is set to 8, and the training optimization algorithm is Stochastic Gradient Descent (SGD) with an initial learning rate of 0.01 and momentum and weight decay of 0.9 and 0.0005, respectively.

### 4.3. Comparison Experiments

To validate the efficacy of YOLO-HVS in infrared small target detection under complex conditions, we conduct comparative experiments on two datasets: the public DroneVehicle dataset and our self-built DroneRoadVehicles dataset (containing 1028 long-range infrared images with occlusions). Four methods are evaluated: Faster R-CNN [31], YOLOv8, YOLOv11 [32], and our YOLO-HVS with MultiSEAM and C2f_DWR modules.

As shown in Table 3 (DroneVehicle dataset), YOLO-HVS achieves state-of-the-art performance with 83.4% mAP50 (1.1% higher than YOLOv8) and 62.5% mAP50-95, while maintaining real-time efficiency (41 FPS). Notably, the proposed MultiSEAM effectively suppresses background interference, reflected in the highest recall (0.787) and F1-score (0.797). Although YOLOv11 achieves lower computational costs (6.3 GFLOPs), its accuracy (82.8% mAP50) is inferior to our method, demonstrating the superiority of our multi-scale feature fusion strategy.

On the DroneRoadVehicles dataset (Table 4), YOLO-HVS further validates its robustness in occlusion scenarios, reaching 97.8% mAP50 (0.7% improvement over YOLOv8) and 64% mAP50-95. The C2f_DWR module’s dilation-wise residual structure enhances multi-scale target detection, as evidenced by the highest recall (0.962) despite complex backgrounds. Visualization results confirm that YOLO-HVS outperforms competitors in low signal-to-noise ratio scenarios.

### 4.4. Ablation Experiments

The proposed framework integrates two key components for infrared small target detection: (1) the MultiSEAM attention mechanism for cross-scale feature enhancement and (2) the C2f_DWR module for dynamic receptive field adaptation. Systematic ablation studies (Table 5) quantitatively demonstrate their individual contributions to detection performance improvement.

As demonstrated in Table 5, on the DroneVehicle dataset, MultiSEAM and C2f_DWR improve mAP50 by 0.4% (from 0.823 to 0.827) and 0.2% (from 0.823 to 0.825), respectively, compared to baseline YOLOv8. The mAP50-95 metric shows corresponding improvements of 0.3% (0.616→0.619) and 0.2% (0.616→0.618). Our proposed method achieves more significant enhancements on the custom dataset (ours) with combined modules delivering 0.7% mAP50 improvement (0.971→0.978).

Notably, the C2f_DWR module demonstrates dual benefits—while improving detection accuracy, it reduces computational complexity by 3.7% in GFLOPs (8.1→7.8) and model parameters by 9.7% (3.1 M→2.8 M), confirming its lightweight characteristics. This enables our model to maintain baseline efficiency while achieving superior detection performance.

### 4.5. Visualization of Results Analysis

**Figure 7:**
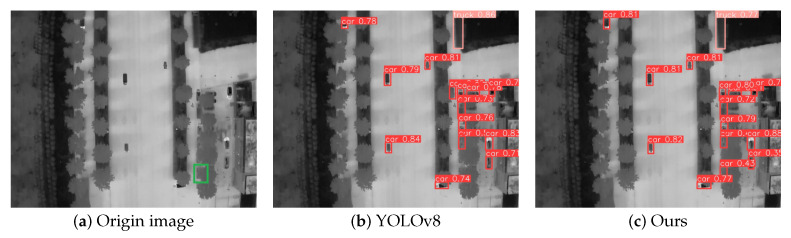
Comparison of small-scale car target detection.

**Figure 8:**
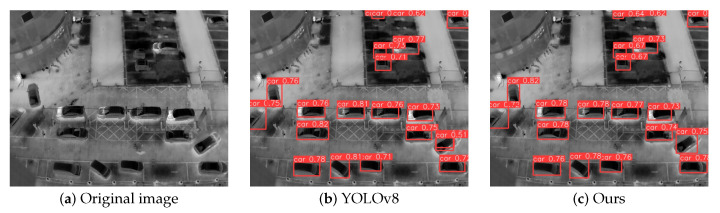
Comparison of standard-scale car target detection.

**Figure 9:**
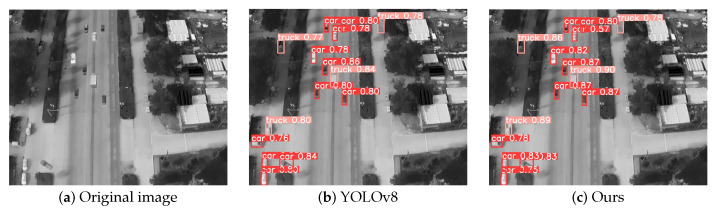
Truck target detection comparison.

To qualitatively evaluate the performance superiority of our approach in detecting infrared small targets under challenging conditions—particularly for multi-scale objects amidst complex background clutter—we conducted visual comparisons using representative samples from the DroneRoadVehicles test set. As demonstrated in Figure 7, Figure 8 and Figure 9, each case study presents: (a) the original infrared input, (b) detection outputs from the YOLOv8 baseline, and (c) predictions generated by our YOLO-HVS framework.

First, in the scenario shown in Figure 7, the core challenge lies in effectively detecting a small-scale vehicle target that is heavily interfered with by road thermal radiation noise and tree trunks (as indicated by the green bounding box in the original Figure 7a). The baseline model YOLOv8 (Figure 7b) failed to identify this target, exhibiting significant false negatives. This reflects the limitations of the original model in handling small targets and suppressing complex background noise. In contrast, our method (Figure 7c) successfully detected the target. This significant improvement is clearly attributed to the integrated MultiSEAM attention mechanism. This module can adaptively focus on critical small target regions, enhancing the model’s ability to extract and distinguish weak target features in information-sparse and noise-intensive scenes, effectively addressing the issue of small target false negatives.

Furthermore, we examined the model’s performance in detecting standard-sized targets and its confidence output. Figure 8 shows a scene containing clearly visible vehicle targets. Both YOLOv8 (Figure 8b) and our method (Figure 8c) successfully detected these targets, indicating that both methods possess foundational capabilities in identifying and locating standard-sized targets. However, a detailed comparison reveals a key difference: our method’s detection results are generally accompanied by significantly higher confidence levels. As shown in Figure 8c, the confidence values of the vehicle detection boxes are notably and consistently higher than those of YOLOv8 in Figure 8b. This strongly suggests that the C2f_DWR module not only optimizes feature extraction efficiency (as demonstrated by the lightweight characteristics proven in the ablation experiments mentioned earlier), but more importantly, it generates more robust and discriminative feature representations. This enhanced feature representation directly translates into higher prediction confidence for the detector, improving the certainty of the model’s output results, which is crucial for subsequent application scenarios (such as early warning and tracking).

Finally, to validate the model’s generalization ability and consistent improvement for typical target categories, Figure 9 focuses on analyzing a truck detection scenario. As a target with distinct size and appearance features within the vehicle category, the detection results for the truck further validate the model’s applicability. Both YOLOv8 (Figure 9b) and the proposed method (Figure 9c) successfully detected the truck target. Building on the observations from the previous scenario, the confidence scores of the detection bounding boxes output by this method are again significantly higher than those of the baseline model. This case not only reinforces the conclusion of improved confidence scores on another target category but also demonstrates that the combination of the MultiSEAM and C2f_DWR modules has universally enhanced the model’s overall feature extraction capabilities and confidence discrimination, rather than being effective only on specific target types.

Quantitative analysis (see Table 4) further supports the aforementioned visualization conclusions: on a specific test subset that specifically reflects complex background interference, YOLO-HVS improves the key metric mAP50-95 from 0.63 to 0.64 compared to the baseline YOLOv8. Combining visualization and quantitative results, YOLO-HVS enhances small object detection capabilities by integrating the MultiSEAM module and by utilizing the C2f_DWR module to optimize feature extraction and confidence generation, significantly improving the accuracy and reliability of infrared small target detection under complex background noise and multi-scale targets. This is of great value for enhancing the perception robustness and decision confidence of unmanned aerial vehicle (UAV) nighttime road monitoring systems in practical applications.

## 5. Conclusions

This paper presents YOLO-HVS, an enhanced YOLOv8-based algorithm specifically designed to address the problems of background interference and multi-scale feature extraction in infrared small target detection. By integrating the attention mechanism of the human visual system and the multi-scale perceptual characteristics, we innovatively designed the MultiSEAM attention module and the C2f_DWR feature extraction module. MultiSEAM significantly improves the target recognition ability in complex occlusion scenes by joint modeling of multi-scale spatial channels, while the C2f_DWR module effectively enlarges the range of the sensory field by the region-semantic dual residual structure. MultiSEAM significantly improves the target recognition ability in complex occlusion scenes through joint modeling of granularity spatial channels, while the C2f_DWR module effectively enlarges the perceptual field range through the region-semantic dual residual structure, realizing the efficient extraction of multi-scale features. The constructed DroneRoadVehicles dataset covers UAV multi-height shooting scenarios, which provides important data support for infrared small target detection research. Comparison experiments on DroneVehicle and self-constructed datasets show that the proposed method improves the mAP50-95 metrics by 0.9% and 1.0%, respectively, while keeping the model lightweight (parametric count of 7.6M, GFLOPs of 10.8), and ablation experiments further validate the effectiveness of the modules. Future work will explore the dynamic sensory field adjustment mechanism to further enhance the adaptability of the algorithm in extreme scale change scenarios.

## Figures and Tables

**Table 1:** Camera basic parameters.

Indicators	Thermal Camera	Visual Camera
Spectral Band	(8–14) μm	(0.38–0.7) μm
Resolution	640 × 512	3840 × 2160/1920 × 1080
Sensors	Uncooled VOx Microbolometer	1/2 CMOS

**Table 2:** Confusion matrix.

Actual/Predicted	Positive	Negative
**Positive**	True Positive (TP)	False Negative (FN)
**Negative**	False Positive (FP)	True Negative (TN)

**Table 3:** Comparative Experiments on DroneVehicle Dataset.

Method	P	R	F1	mAP50	mAP50-95	GFLOPs	Params (M)	FPS
Faster R-CNN	0.73	0.59	0.653	0.674	–	207	41	–
YOLOv8	0.804	0.778	0.791	0.823	0.616	8.1	3.1	26
YOLOv11	**0.809**	0.782	0.79	0.828	0.622	6.3	2.6	50
Ours	0.807	**0.787**	**0.797**	**0.834**	**0.625**	8.2	5.4	41

**Table 4:** Performance Comparison on DroneRoadVehicles Dataset.

Method	P	R	F1	mAP50	mAP50-95
Faster R-CNN	0.887	0.78	0.83	0.823	–
YOLOv8	0.931	0.96	**0.945**	0.971	0.63
YOLOv11	**0.933**	0.957	**0.945**	0.975	0.638
Ours	0.926	**0.962**	0.944	**0.978**	**0.64**

**Table 5:** MultiSEAM and C2f_DWR ablation experiments.

Dataset	MultiSEAM	C2f_DWR	mAP50	mAP50-95	GFLOPs	Params (M)
DroneVehicle			0.823	0.616	8.1	3.1
	✓		0.827	0.619	8.6	5.6
		✓	0.825	0.618	7.8	2.8
	✓	✓	**0.834**	**0.625**	8.2	5.4
DroneRoadVehicles			0.971	0.630	8.1	3.1
	✓		0.974	0.639	8.6	5.6
		✓	0.972	**0.642**	7.8	2.8
	✓	✓	**0.978**	0.640	8.2	5.4

## Data Availability

The original contributions presented in this study are included in the article. Further inquiries can be directed to the corresponding author.
